# Clinical predictors and management for radial artery spasm: an Australian cross-sectional study

**DOI:** 10.1186/s12872-023-03042-z

**Published:** 2023-01-18

**Authors:** Elizabeth Curtis, Ritin Fernandez, John Khoo, James Weaver, Astin Lee, Liz Halcomb

**Affiliations:** 1 Faculty of Science, Medicine and Health, University of Wollongong, Building 41 Office 220, Northfields Ave, Keiraville, NSW 2500 Australia; 2 Centre for Research in Nursing and Health, St George Hospital, 28 Gray Street, Kogarah, NSW 2217 Australia; 3 Cardiology Department, The Wollongong Hospital, Crown Street, Wollongong, NSW 2500 Australia; 4 Cardiology Department, St George Hospital, Grey Street, Kogarah, NSW 2217 Australia

**Keywords:** Coronary angiogram, Radial approach

## Abstract

**Introduction:**

The transradial approach for coronary artery catheterisation has increased in popularity compared to the transfemoral approach for patients undergoing percutaneous coronary interventions. However, radial artery spasm continues to be a major complication of the procedure. Current management strategies vary concerning radial artery spasm and there is limited evidence of practice in the Australian context.

**Aim:**

To identify the predictors of radial artery spasm and the medications used for its prevention and management.

**Methods:**

A descriptive cross-sectional study was carried out over a three-month period in two tertiary hospitals in NSW, Australia. A self-administered pre-procedural survey was completed by patients undergoing coronary artery catheterisation. This survey collected socio-demographic data and assessed anxiety using the Spielberger State-Trait Anxiety Inventory. Procedural data, including length of procedure, equipment used, occurrence of radial artery spasm, and medications given, were collected post-procedure by the interventionalist.

**Results:**

Of the 169 participants, over half were male (59.8%) and aged 66 years or older (56.8%). Radial artery spasm was reported in 24 (14.2%) participants. Rates of spasm were significantly higher among females (66.6%, *p* = 0.004), those aged under 65 years (62.5%, *p* = 0.001) and those who reported a medical history of anxiety (33.3%, *p* = 0.0004). There were no significant differences in State and Trait anxiety scores among those who had RAS and those who did not. Logistic regression identified younger age as the only statistically significant predictor of RAS (OR 0.536; 95% CI 0.171–1.684; *p* = 0.005).

To prevent radial artery spasm most patients received midazolam (n = 158; 93.5%), nitrates (n = 133; 78.7%) and/or fentanyl (n = 124; 73.4%) prophylactically. Nitrates were the most frequently administered medication to treat radial artery spasm (78.7%).

**Conclusion:**

This study highlights that there is a need to develop a clearer understanding of the predictors of RAS, as identifying patients at risk can ensure prophylactic measures are implemented. This study identified nitrates as the preferred vasodilator as a preventative measure along with the use of sedation.

## Introduction

Percutaneous coronary angiography is used for the diagnosis and treatment of coronary artery disease. This procedure allows interventionalists to view the coronary arteries and identify blockages that may be reducing blood flow to the heart muscle. Percutaneous coronary interventions (PCI) involve the insertion of a stent in a blocked coronary artery to revascularise the heart muscle. Advances in PCI techniques have resulted in the increasing use of this treatment modality, thereby reducing the need for coronary artery bypass surgery [1]. This results in significant improvements in patient outcomes and a reduction in health and personal costs [1].

Historically, the femoral artery was the main access site for angiography [2]. However, with improved technologies, other access points have become feasible options, including the radial, distal radial and ulnar arteries [3]. Evidence from a systematic review of randomised control trials found that the transradial approach (TRA) was associated with a decrease in short-term net adverse clinical events, reduction in cardiac death [4, 5], lengths of stay, access site complications [6], bleeding, short term mortality and increased patient satisfaction [7]. Given the benefits, the European Society of Cardiology guidelines for the management of ACS now indicates that TRA should be used for most patients under most circumstances as the preferred route of access (Class 1 recommendation) [8].

Despite the benefits associated with TRA, international rates of uptake of the approach have varied and reportedly range from 16.1% in the United States [9] to 73% in Italy [10]. The variable uptake in rates has been associated with risks and complications associated with TRA, limited learning opportunities and a prolonged learning curve for interventionalists [8]. The risks and complications associated with TRA are multifactorial. Firstly, patients can be exposed to a significantly increased fluoroscopy time and so receive a larger radiation dose when compared to transfemoral procedures. This radiation dose can also be significantly increased depending on the complexity of the procedure [7, 11]. The main complication associated with TRA is radial artery spasm (RAS) [12], which has been reported to occur in some 30% of TRA procedures [13–15]. RAS is the sudden constriction and narrowing of the radial artery, leading to difficulty in the advancement of the catheter and potentially procedural failure [16]. Procedural failure requires cross-over to an alternate access site resulting in longer procedural time and increased risk of complications associated with the alternate access site [7, 15].

Various factors including patient demographics, the presence of cardiovascular risk factors, anatomy of the radial arteries and procedural factors have been reported to increase the risk of RAS [12, 17–20]. Patient demographics linked to higher rates of RAS include female gender, increased age, smaller height, and lower weight [12, 17, 18]. Cardiovascular risk factors identified to increase the occurrence of RAS include hypertension, smoking, and anxiety [17, 19, 20]. Additionally, procedural factors, such as more than one radial puncture attempt or insertion of a ≥ 7F sheath have also been reported to predict RAS [18]. Based on the various factors that increase the risk of RAS, a predictive score for RAS has been developed. This score includes body mass index (BMI), height, hypertension, current smoking status, and peripheral artery disease [17, 18, 21]. The predictive scores enable the implementation of strategies to prevent the occurrence of RAS. Despite the presence of this scoring algorithm, much of the data about RAS and TRA have been drawn from international research. Limited research has been undertaken in the Australian context to understand the predictors of RAS and any variations in practice between countries.

Various prevention strategies including medications and specialised procedural equipment have been advocated to reduce RAS. Medications used include vasodilators, calcium channel blockers, sedation and analgesia, or a cocktail of these medications [22–24]. While a systematic review found that vasodilatory medications reduce the occurrence of RAS, there is insufficient evidence to identify a superior medication, dose or combination [25]. Prevention strategies related to specialised equipment include the use of hydrophilic equipment, special 6-in-5F sheaths, keeping catheter exchanges to a minimum and avoiding cold intra-arterial injections [26]. These strategies are designed to reduce friction and stimulation of the arterial wall subsequently reducing the occurrence of spasm. To date, however, there has been limited exploration of the practices around RAS prophylaxis in the Australian context.

This study is part of a larger study, the AnxieTy, health Literacy and radial Artery Spasm (ATLAS) study, which aimed to identify the association of anxiety and health literacy concerning RAS during transradial coronary angiography.Given the limited evidence in the Australian context about RAS, this study sought to identify predictors, prevention and management of RAS and explore practices concerning TRA for coronary artery catheterisation in Australia. Developing a baseline understanding of practices is important to inform practitioner learning needs specific to each environment. Promoting evidence-based best practice has clear benefits in improving outcomes and ensuring responsible use of finite health resources.

## Methods

This descriptive cross-sectional study was undertaken over a three month period in the cardiac catheterisation laboratories of two metropolitan tertiary hospitals in New South Wales, Australia. At the time of the study both cardiac catheterisation laboratories carried out over 35 radial catheterisation procedures a week. Data collection combined a self-administered survey completed by patients before their procedure and a data sheet completed by the interventionalist following the procedure.

### Participants and recruitment

Participants were outpatients referred for coronary angiography with or without PCI. People were included in the study if they were aged over 18 years and able to provide written consent. Exclusion criteria were people with acute coronary syndrome, hospital inpatients, or those unable to speak or read sufficient English to consent or complete the survey. People with acute coronary syndrome and hospital inpatients were excluded as these characteristics would increase anxiety levels and thus create a bias towards RAS. Patients were invited by a research assistant to participate in the study once they had been admitted and prepared for their procedure. Using a margin of error of 3%, a 95% confidence level, and 300 as the population size, a required sample size of 169 was determined.

### Data collection tools

A survey tool was developed by the researchers based on current evidence on RAS predictors and feedback from interventionalists. A hard copy survey was given to participants to complete before their procedure by the registered nurse after consent was obtained. The survey tool consisted of two sections, the first section explored socio-demographic data, including age, gender, marital status, education, medical history (including history of anxiety) and current medications. The second section measured anxiety using the 40-item Spielberger State-Trait Anxiety Inventory (STAI) [27], a tool used widely to evaluate anxiety [28–31]. The STAI combines state anxiety (20 items), which is anxiety occurring in a current situation, and trait anxiety (20 items), which is anxiety that occurs on most days. Items are scored on a 4-point Likert scale from 1 ‘Not at all’ to 4 ‘Very much so’. A total score is calculated for each scale. A state anxiety score of 36 and over was considered high state anxiety, while a trait anxiety score of 35 and over was considered high trait anxiety [32]. The internal consistency coefficients of the STAI range from 0.86 to 0.95 and test–retest reliability coefficients range from 0.65 to 0.75 [32].

Post-procedural data were collected using a data sheet completed by the interventionalists. The data sheets were matched to patient surveys using patient codes. Data collected post-procedure included medications and equipment used during the procedure, the occurrence of RAS and other complications and the time taken for the procedure. The presence of RAS was documented if there was restricted movement of the catheter and forearm pain [25] and spasm was confirmed with radial artery imaging.

### Ethics

Ethical approval was obtained from the University (Approval No XXX) and Hospital Human Research Ethics Committee (Approval No XXX). Participants were informed that all data would be de-identified and aggregated, and that non-participation would not affect their care. Written consent was gained from patients before completing the survey. Confidentiality was maintained using numerical unique identifiers. The list of numbers and patient names were kept separately by the lead researcher.

### Analysis

All hard copy data were entered into SPSS V 25 [33] for analysis by an independent research assistant and data was cross checked by two members of the research team. Continuous data were presented as means/standard deviation and categorical data as frequencies and percentages. Age was dichotomised into less than 65 years and 66 years and over. Univariate analysis was used to identify associations between demographic and clinical factors and the occurrence of RAS. Factors that were significant in univariate analysis were then subject to logistic regression using the Hosmer–Lemeshow test [34]. The Hosmer–Lemeshow test correctly fitted the data (χ^2^ = 3.344; sig. = 0.911) indicating that the overall model fit is good. A *p* > 0.05 was considered statistically significant.

## Results

### Patient characteristics

One hundred and ninety-five participants were recruited during the study period, however, data on RAS occurrence were missing for 26 participants (13.3%), leaving 169 (86.7%) participants for inclusion in the analysis. While the registered nurses were asked to collect data on the eligible participants who declined to participate, these data were not always collected due to time pressures and administrative workload. Therefore, it is not possible to calculate a response rate.

The majority of participants were male (n = 101; 59.8%), aged 66 years or over (n = 96; 58.2%), and were non-smokers (n = 149; 90.4%). Reported previously-diagnosed conditions included high cholesterol (n = 77; 45.6%), hypertension (n = 73; 43.2%), heart disease (n = 44; 26%) and anxiety (n = 18; 10.7%) (Table 1). Overall interventionalists reported administering heparin (n = 158; 93.5%), vasodilators (n = 134; 79.3%), and sedatives (n = 155; 91.7%) to participants.

**Table 1:** Patient characteristics and univariate analysis

	All patients (n = 169)	Patients with radial artery spasm n (%)	*P* value*
*Gender*			
Female	64	16 (66.6)	0.003**
Male	101	8 (33.3)	
*Age*			
Age ≤ 65 years	58	15 (62.5)	0.001**
Age ≥ 66 years	96	6 (25)	
Smoker	16	2 (8.3)	1.000
*Medical history*			
Anxiety	18	8 (33.3)	< 0.001**
Depression	21	4 (16.6)	0.506
Diabetes	39	7 (29.2)	0.441
High blood pressure	73	9 (37.5)	0.658
High cholesterol	77	9 (37.5)	0.508
Family history heart disease	42	7 (29.2)	0.614
Heart disease	44	10 (41.7)	0.078
Stroke	8	2 (8.3)	0.317
Peripheral vascular disease	6	1 (4.2)	1.000
*State anxiety score*			
Score < 36	78	7 (29.2)	0.118
Score ≥ 36	90	16 (66.6)	
*Trait anxiety score*			
Score < 35	85	10 (41.7)	0.505
Score ≥ 35	82	13 (54.2)	
*Medications*			
Nitrates	133	20 (83.3)	0.788
Calcium channel blocker (verapamil)	1	0	1.000
Midazolam	153	24 (100)	0.360
Fentanyl	124	19 (79.2)	0.621
No medications	7	0	0.595
*Angiogram*			
Diagnostic only	120	20 (83.3)	0.601
Angioplasty	34	4 (16.7)	
Duration		45.7 (19.3)	0.017**

*Statistical analysis carried out using Chi-square test

**Statistically significant (*p* < 0.05)

The mean state and trait anxiety scores were 36.47 (SD = 11.59) and 35.37 (SD = 9.83) respectively. Over half (n = 90; 53.3%) of the participants were classified as having high state anxiety, while less than half (n = 82; 48.5%) were classified as having high trait anxiety. Cronbach’s alpha was 0.889 for state anxiety and 0.894 for trait anxiety, demonstrating high reliability.

### Procedural characteristics

Procedures were performed by twelve interventionalists and eleven advanced trainees. Most (n = 114; 69.1%) procedures were carried out by advanced trainees, however, 22 (13.3%) procedures were carried out by interventionalists and 21 (12.7%) procedures were carried out by both the advanced trainee and interventionalists.

Just over three-quarters of participants had a diagnostic angiogram (n = 120; 77.5%) (Table 1). Two (4.1%) participants had the procedure through the left radial artery, while the remainder were through the right radial artery (n = 162; 95.9%). In most procedures (n = 155; 91.7%) a 6-French sheath was used, while 4.7% (n = 8) had a 5-French sheath, the remaining were not recorded. Two or fewer catheters were used in 101 cases (61.9%) and while over a third of procedures (n = 62; 38.0%) had three or more catheters, the remaining six participants did not have the number of catheters recorded. The mean duration of the procedures was 35.5 min (SD 22.3 min).

### Adverse events

Complications were experienced by 32 (18.9%) participants. These were RAS (n = 24; 14.2%); haematoma (n = 4; 2.4%), artery dissection (n = 1; 0.6%), infection (n = 1; 0.6%), pseudo-aneurysm (n = 1; 0.6%) and brachial perforation (n = 1; 0.6%). Rates of RAS were not significantly different between procedures carried out by a trainee alone (n = 11; 10.5%) compared to a consultant alone (n = 3; 13.6%). Crossover to the femoral artery during the procedure was required in 8 (4.7%) participants in total, five (62.5%) of these were because of RAS.

### Predictors of RAS

Univariate analysis demonstrated a significantly higher incidence of RAS in participants aged under 65 years compared to those over 65 years (*p* = 0.001), those with a medical history of anxiety (*p* = 0.0004), female participants (*p* = 0.004) and those with an increased length of procedure time (*p* = 0.017). Multiple logistic regression identified younger age as a statistically significant predictor of RAS (OR 0.917; 95% CI 0.867–0.970; *p* = 0.003) (Table 2).

**Table 2:** Multivariate logistic regression analysis

Factor	*P* value	OR	95% CI for EXP(B)	
			Lower	Upper
Gender	.168	.460	.153	1.388
Age	.003	.917	.867	.970
History of anxiety	.090	3.554	.821	15.376
Constant	.080	87.285		

### Prophylactic medications for RAS

To prevent RAS, most patients received midazolam (n = 153; 90.5%), nitrates (n = 133; 78.7%) and/or fentanyl (n = 124; 73.4%) prophylactically. Only 19 participants (11.5%) received a single medication. Over half (n = 108; 65.5%) of participants received a combination of nitroglycerin, fentanyl and midazolam, while combinations of two medications were administered to 35 (21.2%) participants. Five participants (3%) received no medications before the procedure. There was no statistical significance in relation to RAS and medications used (*p* > 0.05).

### Medications to treat RAS

Of the 24 participants who experienced RAS, 9 (37.5%) participants were administered nitrates to resolve the spasm, while 6 participants (25%) received additional midazolam, 5 (20.8%) participants received no treatment, and 4 participants (16.7%) received fentanyl.

## Discussion

The TRA is becoming increasingly popular in coronary angiography, however, RAS continues to be a major complication of the procedure. Given the limited research in the Australian context assessing the predictors, prevention, management and prevalence of RAS, this study provides new insight into current practice in the Australian context. The prevalence of RAS seen in this study (14.2%), is consistent with international rates of RAS reported in the literature [14]. While several predictive factors are congruent with some findings reported internationally, there remains a lack of consensus between studies. This demonstrates the need for ongoing monitoring of RAS prevalence and predictors to promote a shared understanding of factors that increase the risk of RAS.

This study highlighted an association between age and RAS, with RAS occurring more frequently in younger patients. This is consistent with Trilla, Freixa [16] who found that patients who experienced RAS were generally younger. The lower incidence of RAS in older adults may be due to the processes of muscular denervation and endothelial dysfunction that have been associated with aging [16]. However, as other studies have identified older age as a predictor of RAS [17], further research is required to confirm the direction of this association.

This study found a relationship between those who had a self-reported medical history of anxiety and RAS, although did not find an association between RAS and preprocedural anxiety. This is in contrast with previous studies that have found a statistically-significant correlation between RAS and state anxiety [19, 20]. This indicates that those with prolonged levels of anxiety may be more susceptible to RAS compared to those who have a sudden spike in anxiety due to the impending procedure.

Consistent with other studies [12, 17, 18], this investigation found that the female gender was a predictor of RAS. Mong, Duggan [35] identify that the radial arteries of females are more sensitive to vasoconstrictors, while being less sensitive to vasodilators when compared to males. Additionally, females have smaller radial arteries, impacting the radial artery to sheath ratio, which increases the risk of RAS [17]. Some operators have a preference for starting with smaller-bore catheters for females because of these factors.

In this study, nitroglycerin was the most common vasodilator used prophylactically while midazolam was the most common sedative used to reduce RAS. The preference for these drugs is inconsistent with previous international studies, which have demonstrated a preference for either verapamil (9) or a cocktail of verapamil and nitroglycerin(21).. In this study, verapamil was only administered to one patient which is inconsistent with international practice. This could be due to the availability of the drug or patient contraindications for the use of verapamil. Best practice documents encourage the use of vasodilatory medications to reduce the incidence of RAS, while acknowledging the choice of medication is up to the operator [8, 13, 14]. Despite the differences in medication choice, the similar prevalence of RAS demonstrates that it is unlikely that the variation in drug administration impacted care.

The variation in dosages of prophylactic medications seen in this study is consistent with the literature [25]. In this study, no single vasodilatory medication was administered to over 14% of patients, indicating the wide variability in operator preferences. Both Hamon, Pristipino [13] and Mason, Shah [8] support the use of pre-procedural sedation to reduce the risk of RAS, noting that decreased discomfort and anxiety may assist in reducing the stimulation of neural pathways and arterial vasoconstriction. In this study, greater than 90% of patients received midazolam and/or fentanyl, a typical sedation cocktail administered before the procedure. However, the inconsistecies in prophylactic medications highlight the need for further work to ensure that best practice is implemented in the clinical setting.

This study found nitroglycerin to be the preferred medication to relieve RAS once it occurred followed by midazolam. Current evidence based guidelines identify additional spasmolytic therapy and analgesic administration when spasm occurs [14]. Spasmolytics include calcium channel blockers, such as verapamil, while suggested analgesics include midazolam, morphine or fentanyl. Although nitroglycerin does not fall under the category as a spasmolytic, it does have a vasodilating impact on the artery resulting in reports of sublingual or intra-arterial nitroglycerin as options to resolve RAS [36]. The use of intra-arterial nitroglycerin is common for other reasons apart from RAS in coronary procedures, so familiarity with this medication and its effects may be a reason this is most commonly used to relieve RAS.

### Strengths and limitations

The major strength of this study was that it was undertaken at two different sites and included a sizeable number of clinicians undertaking the procedure to reduce the impact of personal preference. Additionally, the combination of patient and procedure data broadened the dataset. Using a validated instrument to measure patient anxiety ensured reliable and valid measurement.

Despite these strengths, there are several limitations. This study focused on procedural data, as a result some variables that may impact on the outcomes were not included (e.g. nutritional status, body mass index). Further research on the impact of these factors iswarranted. Although data were collected across three months, the sample size is modest. Due to factors beyond our control data about those who were excluded or did not consent was not always collected. The exclusion of people who did not have sufficient English language to consent or complete the survey restricts the cross-cultural generalisability of the study. Previous patient experience with angiography or associated procedures, that could impact on anxiety levels, was not evaluated. Additionally, medical history of anxiety was self-reported and therefore some participants may not have disclosed their medical history accurately.

## Conclusion

Australian rates of RAS are consistent with those reported internationally. While this study demonstrated predictors for RAS to be younger age, females and medical history of anxiety, findings across the literature are not consistent. While the medication regimes used prophylactically in this study showed some variation from those reported internationally, they did not lead to significant differences in RAS prevalence. These international differences highlight the need for ongoing evaluation of large-scale procedural data internationally to achieve clarity around predictive factors and best practice. By understanding these data clinicians will be better able to screen patients before their procedure and reduce the prevalence of RAS.

## Data Availability

The datasets used and/or analysed during the current study are available from the corresponding author on reasonable request.
