# Correction: Clinical predictors and management for radial artery spasm: an Australian cross‑sectional study

**DOI:** 10.1186/s12872-023-03119-9

**Published:** 2023-02-17

**Authors:** Elizabeth Curtis, Ritin Fernandez, John Khoo, James Weaver, Astin Lee, Elizabeth Halcomb

**Affiliations:** 1 Faculty of Science, Medicine and Health, University of Wollongong, Building 41 Office 220, Northfields Ave, Keiraville, NSW 2500 Australia; 2 Centre for Research in Nursing and Health, St George Hospital, 28 Gray Street, Kogarah, NSW 2217 Australia; 3 Cardiology Department, The Wollongong Hospital, Crown Street, Wollongong, NSW 2500 Australia; 4 Cardiology Department, St George Hospital, Grey Street, Kogarah, NSW 2217 Australia

**Correction: BMC Cardiovascular Disorders (2023) 23:33** **https://doi.org/10.1186/s12872-023-03042-z**

Following the publication of the original article [1], the author name Elizabeth Halcomb was incorrectly written as Liz Halcomb.

Also, in this article the Ethics statement was incorrectly given as ‘Ethical approval was obtained from the University (Approval No XXX) and Hospital Human Research Ethics Committee (Approval No XXX).’ but should have been ‘Ethical approval was obtained from the Hospital Human Research Ethics Committee (Approval No 16/234)’.

The original article has been corrected.
